# Unsupervised acquisition of idiomatic units of symbolic natural language: An n-gram frequency-based approach for the chunking of news articles and tweets

**DOI:** 10.1371/journal.pone.0234214

**Published:** 2020-06-08

**Authors:** Dario Borrelli, Gabriela Gongora Svartzman, Carlo Lipizzi

**Affiliations:** School of Systems and Enterprises, Stevens Institute of Technology, Hoboken, New Jersey, United States of America; University of Vermont, UNITED STATES

## Abstract

Symbolic sequential data are produced in huge quantities in numerous contexts, such as text and speech data, biometrics, genomics, financial market indexes, music sheets, and online social media posts. In this paper, an unsupervised approach for the chunking of idiomatic units of sequential text data is presented. Text chunking refers to the task of splitting a string of textual information into non-overlapping groups of related units. This is a fundamental problem in numerous fields where understanding the relation between raw units of symbolic sequential data is relevant. Existing methods are based primarily on supervised and semi-supervised learning approaches; however, in this study, a novel unsupervised approach is proposed based on the existing concept of n-grams, which requires no labeled text as an input. The proposed methodology is applied to two natural language corpora: a Wall Street Journal corpus and a Twitter corpus. In both cases, the corpus length was increased gradually to measure the accuracy with a different number of unitary elements as inputs. Both corpora reveal improvements in accuracy proportional with increases in the number of tokens. For the Twitter corpus, the increase in accuracy follows a linear trend. The results show that the proposed methodology can achieve a higher accuracy with incremental usage. A future study will aim at designing an iterative system for the proposed methodology.

## Introduction

Symbolic sequential data are generated every day in huge quantities and include textual data published on the web, data produced by biomedical sensors on human bodies, financial market indexes, and sequences of DNA. Extracting patterns of related units can help those disciplines involved in the rules that generate them. In this study, the focus is on a specific type of raw symbolic sequential data, namely, natural language textual data.

The unsupervised identification of idioms in raw natural language data requires an unsupervised acquisition of varied elements embedded in a language such as grammar, syntax, semantics, phonetic, phonology, semiotic, and morphology. One system completely capable of achieving this is the human brain. Debate is still ongoing regarding whether a machine can learn such rules from plain text without supervision.

Isolating the syntax would make the learning task easier for a human child, whereas learning the syntax requires information on the semantic, acoustic, and visual elements, all of which contribute to language acquisition [1]. Chomsky [2] introduced the theory of universal grammar, hypothesizing that humans have a language acquisition device (LAD) that allows them to learn the grammar and syntax of any language. According to Chomsky’s hypothesis, a human child has the necessary means to speak but does not have yet the experience that allows the child to compile complex sentences together (a so-called “poverty of stimulus”).

The process of creating learning models able to parse languages in an unsupervised fashion has received considerable attention from many scholars in the fields of natural language processing (NLP), natural language understanding (NLU), and computational linguistics (CL), among others in which sequential data are relevant. Although syntax and grammar induction are a simplification of the human process when acquiring a language, the widespread approach of isolating the syntax is based on representational modularity theory, which states that the human mind encodes information in separate layers and formats, and that syntax is one of these layers [3]. By contrast, it is unclear whether additional layers of semantics and phonology [4, 5] can help improve the text chunking process for a machine.

Most supervised approaches for learning syntax use hand-parsed sentences on large tree-banks, which are corpora analyzed and annotated with the help of domain experts. The NLP community has recently been making extensive use of an unsupervised method for generating a distributional representation of text units based on an artificial neural network, called text embedding. The units applied by the embedding models can vary from the scale (see the article by Febres et al. [6] for the concept of scale) of characters, to the scale of words, up to the paragraph and document levels. Many NLP tasks have been improved with the application of these new vectorization techniques, including text chunking [7–10]. Parse trees, syntax annotations, and part-of-speech (PoS) tagging are not necessary for training these embedding models. In fact, such models require a set of textual units as an input without having a specified syntactic role that each textual unit must have. By contrast, most of the existing approaches for shallow parsing are based on syntax annotations. The use of syntactical annotated treebanks has been the primary option of many supervised text chunking methods, typically demonstrating a high accuracy. However, the accuracy of such supervised-learning-based chunkers degrades widely when applied to new texts, new genres, and new languages [11]. The unsupervised learning of natural language is a difficult but basic research problem, as highlighted by Klein and Manning [11]. After numerous years of effort, the accuracy of the results is still unacceptably low when compared against the high accuracy of semi-supervised and supervised approaches. However, the supervision provided to these models leads to a lack of generalization, particularly among the contexts in which natural language deviates from traditional syntax rules (e.g., social media). Unlike other works that intend to induce a tree structure that governs natural language, this work generalizes to the simplest detection of sequences of symbolic units that occur with a certain frequency. In this way, the problem of domain adaptation is addressed because it is possible to extend the method to other diverse sequential data. This work illustrates an intuitive unsupervised approach evaluated on two different types of corpora: news and social media text.

The main contribution of this research is the proposed methodology. This methodology is based on n-gram theory to create text chunking classification without any tags, i.e., unsupervised learning. A chunk is a unit of text that refers to a specific syntactical phrase, which can be a noun-phrase or a verb-phrase. The proposed methodology has the flexibility of accepting text in different languages and from different sources, without relying on any tags or pre-classification. The motivation in creating this methodology lies in the possibility of improving any NLP task that currently requires labels to compute, even providing the possibility of a real-time text analysis. This can have an impact on understanding one’s perception from a text, creating narratives from text, pursuing security against cyber-threats, and even improving artificial natural language systems designed to have a positive impact on society.

The remainder of this paper is organized into five main sections. The current section introduces the problem, the proposed approach, and the motivation behind it. The next section, describes previous studies on semi-supervised, supervised, and unsupervised approaches. The following section describes in detail the methodology proposed for implementing and evaluating unsupervised text chunking. The methodology applied to two different corpora is then described by measuring and comparing their accuracy and performance. These experimental results are then described in the next section. Finally, some concluding remarks are provided along with limitations and areas of future study.

## Related studies

### Supervised and semi-supervised approaches

Supervised or semi-supervised text chunking models typically rely on a gold standard annotated corpus used for training a machine learning or deep learning model. The Conference on Computational Natural Language Learning (CoNLL) created a shared task specifically for text chunking [12]. Since 2000, the CoNLL-2000 corpus has been the default standard for benchmarking between different text chunking methodologies.

The CoNLL-2000 corpus consists of 8,936 training sentences and 893 test sentences extracted from Wall Street Journal articles from the English Treebank. The corpus has been manually annotated following the Begin-Inside-Outside (BIO) labeling standard, which assigns a label to each token of the corpus. Distinctively, in any given sentence, single-word-tokens are extracted and annotated with one of the BIO labels. The “B” in BIO demonstrates that the token is the first word of the chunk, “I” indicates that the token is inside (or at the end) of a chunk, and “O” shows that the token is outside any chunk. Having an annotated corpus leads intuitively to a supervised learning model approach.

Numerous authors have adopted supervised text chunking using Hidden Markov Models (HMM) for predicting PoS tags and forming chunks accordingly to syntactic rules [13–16]. Other groups have approached the same problem using conditional random fields (CRFs) [17–19]. Owing to their specific characteristics, both methods have been extensively adopted for studying different sequential data. A recent research direction has applied supervised text chunking tasks using artificial neural network architectures. Zhai et al. [20] showed successful results when applying a recurrent neural network (RNN), long short-term memory (LSTM), and their variations.

A significant number of studies have adopted pre-trained word embeddings for improving the results of the above-mentioned techniques [21–23]. Pre-trained embeddings are also being used in this field, including bidirectional encoder representations from transformers (BERT) applied by Devlin et al. [24] and embeddings from language models (ELMo) developed by Peters et al. [25]. Both BERT and ELMo have been evaluated for their syntactic recognition capabilities, which can improve the supervised text chunking [26].

In general, a global ranking is assumed for the quality of the available pre-trained embeddings. However, Faruqui et al. [27] and Schnabel et al. [28] found that the accuracy of text chunking methods using pre-trained embeddings does not necessarily reflect the same global rankings. For this reason, different combinations [29, 30] of pre-trained embeddings have been tested for different chunking methods, i.e., from the scale of characters [31] to the scale of words using supervised and semi-supervised approaches [32]. Supervised shallow parsers have become publicly available using software and programming language library solutions [33–35]. Abnik et al. [35] made their chunker available in a Python library called Flair. Other NLP libraries (SpaCy and Natural Language Tool Kit) also provide users with supervised noun-phrase chunkers trained on the CoNLL-2000 corpus. Supervision limits the parsing model to the available annotated corpora, and therefore a supervised approach for text chunking has been used for context biased text with domain- and language-specific annotated corpora: for instance, chunkers for the bio-medical and medical fields [36], chunkers for software engineering [37], Chinese, Thai, and Arabic language chunkers [38–40], and social media text chunkers [41–43]. Table 1 groups the text chunking methods found in the literature, whereas Table 2 shows an overview of supervised approaches for text chunking with their respective performances on specific annotated datasets.

**Table 1:** Approaches from the literature, among which a few are unsupervised.

Authors	Learning Paradigm			Embeddings	Language	Corpus
	Supervised	Semi-supervised	Unsupervised			
[20, 22, 23, 31, 43–45]	✓			✓	English	WSJ news
[37]	✓			✓	English	Biomedical corpus
[41]	✓				Others	Software code
[46]	✓				Others	Thai corpus
[40]	✓				Others	Arabic treebank
[32, 47]		✓		✓	English	WSJ news
[38]			✓		Others	Chinese treebank
[48–50]			✓		English	News

**Table 2:** Remarkable performances of supervised methods.

Authors	Data set	Domain	*F*_*β*=1_	Method
[32]	CoNLL-2000	News	96.98%	GloVe Embeddings + biLSTM + CVT
[31]	CoNLL-2000	News	96.72%	LSTM + Character Embeddings
[51]	CoNLL-2000	News	96.49%	Hidden Markov Model
[22]	CoNLL-2000	News	96.32%	Bi-Language Model
[20]	CoNLL-2000	News	95.86%	LSTM Bi-directional & Pointer Network
[36]	GENIA Corpus	Biomedical corpus	95.70%	Gate Chunker
[47]	CoNLL-2000	News	93.88%	Word embeddings + POS tags supervision
[43]	CoNLL-2000	News	93.50%	RNN + LSTM
[51]	Penn Arabic Treebank	News	93.48%	CRF
[44]	CoNLL-2000	News	93.48%	Support Vector Machine
[23]	Chinese Corpus	Mixed	91.76%	Character Recognition, CRF & RNN
[41]	Alan Ritter	Twitter	86.7%	TwitterNLP

### Unsupervised approaches to text chunking

Several studies on NLP and CL have focused on unsupervised machine learning approaches for learning languages. Some studies have generalized the learning tasks to different natural languages, for example, biological sequences, text or speech [52], and music [53, 54], among others [55], which are represented through sequential symbols [56]. Seginer [57] introduced a model called common cover links (CCL), which learns dependencies in a text (in a tree form) without any previous annotation. Subsequently, Ponvert [48] explored this method and discovered that the performance obtained from unsupervised parsing with the CCL significantly depended on the identification of low-level constituents, i.e., words that compose the lowest leaves of a parse tree.

The identification of low-level constituents is considered a “partial” parsing, because the internal role of each word in a chunk is not really known or taken into consideration. Hence, unsupervised partial parsing is a sub-problem of unsupervised parsing. To this extent, unsupervised partial parsing has been defined as the “unsupervised version of text chunking” by Ponvert et al. [58], who also presented a simple unsupervised bi-gram model comparable to a CCL unsupervised parser. This is an important result for two main reasons: It demonstrates that low-level constituents are the elements that matter for full parsing, and it proves that the type of chunk representations used are closer to supervised text chunking outputs.

A reputable unsupervised grammar induction method was proposed by Klein and Manning [59] and later improved upon by Headden et al. [60] and Cohen [61]. Each of these methods assumes the PoS to be known, as derived from the gold standard annotation. Seginer [57], followed by Ponvert et al. [49], provided grammar induction models for learning from plain text alone without any given manual annotation. The goal of text chunking is to identify the low-level constituents in a text without providing the tree structure of sentences that are typical of dependency or constituency parsing. This issue has been addressed in the past using traditional grammar induction and unsupervised methods. The goal of unsupervised text chunking is the same, with the exception that chunks must be learned without providing any form of supervision (e.g., treebank annotations or human feedback).

Abney [62] highlights the differences between low-level constituents and chunks. A low-level constituent (called a “clump” by Ponvert et al. [58]) has two main differences with a chunk: the labeling and the additional components found in the chunks. In 2000, a shared task on text chunking was introduced by Tjong at al. [12]. This task solved the chunking problem with the help of a dedicated labeled dataset. It is still used as a benchmark corpus, and a high accuracy has been obtained by applying different supervised and semi-supervised learning methods [31, 32]. One of the main advantages of unsupervised text chunking is the independence from the corpus to the chunk-specific domain. In fact, an unsupervised method does not require the manual construction of domain-specific annotations. Because they must be performed by domain experts, the placement of annotations and the building of gold-standard treebanks for a specific domain are time-consuming and labor intensive.

As previously mentioned, it was proven by Mikolov et al. [63] that the performance of supervised methods, which rely on gold annotated treebanks, decays dramatically when applied to other domains or other languages. Mikolov et al. [63] also mentioned that the generation of distributed representations of textual units would be adopted in NLP owing to the improvements they provide to different NLP tasks (e.g., the text classification task by Jang et al. [64]).

Such techniques, including Word2Vec, acquire words or chunks of words as an input and generate vectors as an output. The input units do not need to be tagged with the PoS tags or any other annotation that follows syntax and grammar. However, the input units must be meaningful to obtain a significant vector representation. Hence, embedding models require unsupervised methods for text chunking to produce chunks ready to be processed. An important consideration highlighted by Lightfoot [65] and Cocho et al. [66] was that language changes over time and so do the uses of the same languages. Chunks obtained using a supervised method with an annotated corpus produced 20 years ago may not work properly today. A text published on a social media site may not necessarily reflect the rules defined in a treebank built by a domain expert using supervised training models. This means that the methodology can experience bias when applied to different corpora. There has been a lack of effort regarding unsupervised text chunking (compared to supervised text chunking), which is also known as unsupervised partial parsing. Ponvert et al. developed an initial methodology for text chunking. In addition, Zhu et al. [38] improved on this methodology to create an unsupervised approach for the Chinese language that uses English as a seed language, providing a basic form of supervision. It is evident that there is an opportunity to keep exploring this field of study and develop new unsupervised text chunking techniques in line with previous published approaches [50, 52, 58].

## Materials and methods

**Fig 1:**
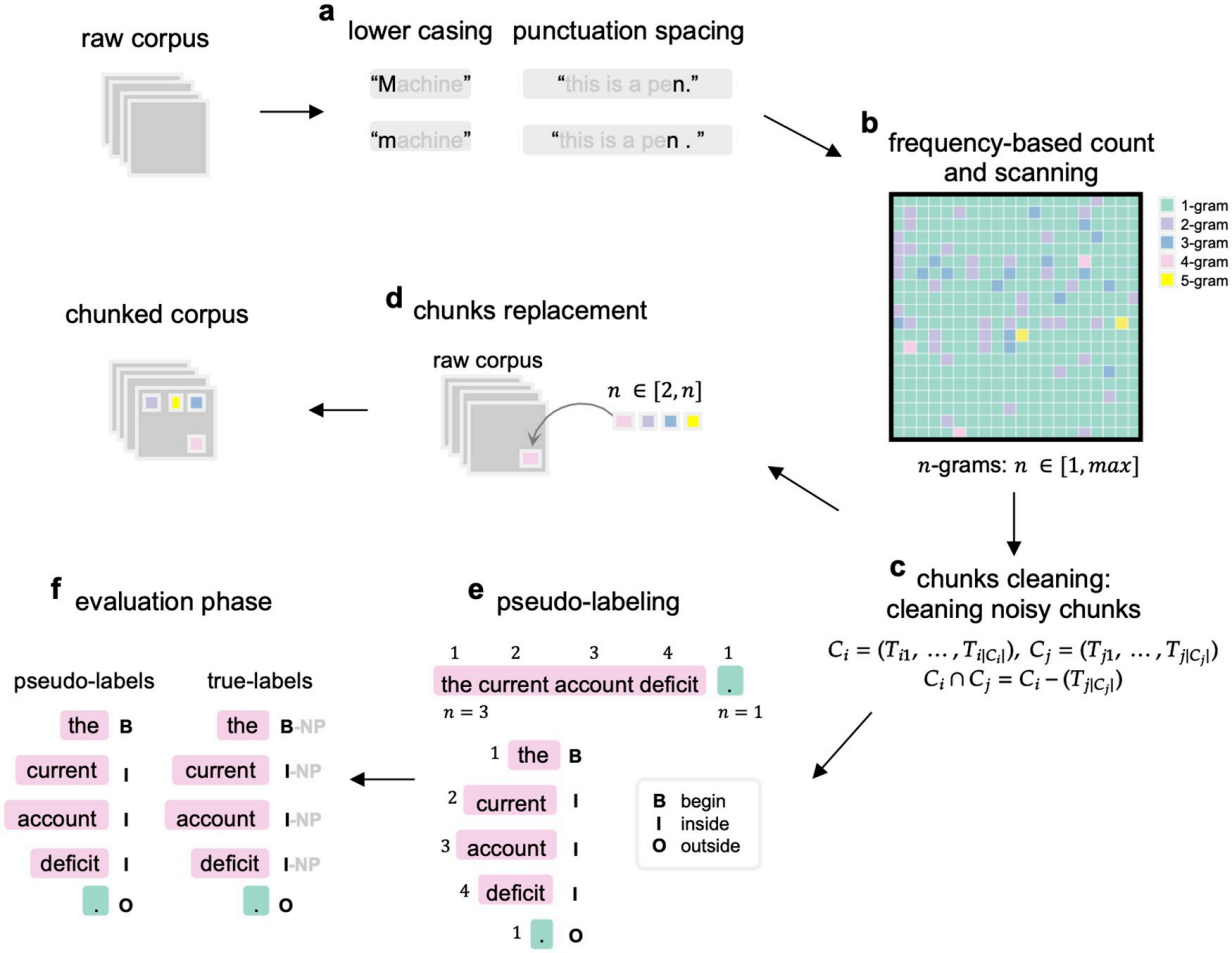
Flowchart of the proposed methodology. Steps in proposed unsupervised methodology for text chunking.

This work involves an analysis and evaluation of a proposed unsupervised method for extracting textual chunks. This method is presented throughout this section, which details every step of the chunking process. Later sections provide an application of the methodology, evaluation measures, and discussions. The proposed unsupervised method for text chunking consists of four sequential steps, as illustrated in Fig 1.

A plain text is required as an input to the chunker. The next steps are followed for the proposed methodology.

### Basic pre-processing (Fig 1a)

This first step is made up of two sub-steps:

1. *Lower casing*—Every character has a lower-case version in the plain text to avoid potential duplicate strings, for example, “Machine” and “machine.” The lower-case text is used as an input to a function separating the punctuation characters from neighboring words. For instance, “this is a pen.” is transformed into “this is a pen.” by adding a space between “pen” and the period. This is executed to avoid further duplicates that may reduce the quality of the final chunks.
2. *Punctuation and spacing*—This sub-step simply implies removing any punctuation symbols (e.g., “?” and “;”), special characters, and spacing that can cause problems when analyzing the text.

### Detection of frequent chunks (Fig 1b)

This step involves calculating the frequency of repeated n-grams for *n* = 2 until *n* = *max*. The maximum value (*max*) of *n* is the length of the longest sequence of tokens repeated at least once in the plain pre-processed text. For every *n*, the chunks with a frequency of greater than 1 are stored in a separate set. The output is a set containing all candidate chunks. An example of this list is as follows: {“machine learning,” “machine learning is,” “machine learning algorithms,” “learning algorithms,”…}. This list of candidate chunks is noisy. In fact, it contains 2-grams and 3-grams that present a common part (e.g., “machine learning,” “machine learning is,” “machine learning algorithms”). Preliminary parameters are computed while scanning the input corpus to explore patterns of co-occurrences of unitary tokens. These parameters are required for a calculation of the frequencies of co-occurring n-grams. In turn, these frequencies are the basis for chunking any corpus. The four parameters can be described as follows:

1. *Total non-unique number of tokens in the corpus* (*T*). For example, considering the small corpus, “text chunking is an important task in NLP,” the total non-unique number of tokens is eight. This value has been calculated by spacing the punctuation, splitting the corpus into tokens using spaces, and counting their resulting number.
2. *The total number of repeated n-grams* is a relevant and related metric. It counts the number of repeated n-grams, i.e., the total number of n-grams with a frequency of greater than 1. In the same example from the first metric, the total number of repeated n-grams is equal to zero because none of the possible n-gram are repeated within the corpus. If the corpus is changed into “Text chunking is an important task in NLP. Text chunking is also known as shallow parsing,” the number of repeated 2-grams is equal to 1 because there is only one n-gram with a frequency of greater than 1. “Text chunking” is repeated twice. This can be calculated through the following procedure. First, the total number of n-grams (*η*) can be represented using Eq 1:
  $$\begin{array}{c} \eta =[\;\sum_{n=2}^{\max}(T+1\;-\;n)\;]+T \end{array}$$ (1)
  The set *τ* of all repeated n-grams can then be written as follows:
  $$\begin{array}{c} \tau \;=\;\{x_{1},x_{2},...,x_{\eta }\} \end{array}$$ (2)
  where *x*_*i*_ is a generic *i*^*th*^ n-gram, and |*τ*| = *η*. By calculating the frequencies of each n-gram in *τ*, it is possible to obtain the following set:
  $$\begin{array}{c} R=\{x_{i}\in \tau \;:\;F_{x_{i}}>1\}\subseteq \tau \end{array}$$ (3)
  where $F_{x_{i}}$ is the frequency of a generic *i*^*th*^ n-gram *x*_*i*_. The cardinality of the set R provides the total number of repeated n-grams (*ρ*):
  $$\begin{array}{c} \rho =|R| \end{array}$$ (4)
3. *The sum of all n-gram frequencies that repeatedly appear when pre-processed*. In the example “text chunking is an important task in NLP. Text chunking is also known as shallow parsing.” This metric, calculated for 2-grams, is equal to 2 because the sum of the repeated bi-gram frequencies is equal to 2. This metric can be formalized through Eq 5:
  $$\begin{array}{c} \varphi =\sum_{x_{i}\epsilon R}F_{x_{i}} \end{array}$$ (5)
4. *The total number of unique tokens*. This metric is equivalent to the total number of unique tokens in the plain pre-processed text, i.e., the total non-unique number of tokens when there are no repeated tokens in the corpus. Because the proposed methodology is dependent on the frequencies of repeated patterns, this metric is an indication of how much the corpus has n-gram repetitions, hence, potential chunks.

### Chunks cleaning (Fig 1c)

This step refers to the automatic cleaning of the list created in the previous step from the incorrect chunks (e.g., “machine learning is”). Consider two candidate chunks *C*_*i*_, *C*_*j*_. Both can be represented as an ordered set of tokens described by Eq 6. The intersection between the two sets of tokens is then calculated to check if the following equivalence is true through Eq 7. In the affirmative case, *C*_*i*_ will be excluded from the final list of candidate chunks only if *T*_*j*|*C*_*j*_|_ is one of the most-frequent 1-grams in the initial corpus, and if it has been typically found to be an English stop word or a punctuation character for an English corpus.

$$\begin{array}{c} C_{i}=(T_{i1},\ldots ,T_{i|C_{i}|}),{\;C}_{j}=(T_{j1},\ldots ,T_{j|C_{j}|}) \end{array}$$ (6)

$$\begin{array}{c} C_{i}\cap C_{j}=C_{i}-(T_{j|C_{j}|}) \end{array}$$ (7)

where |*C*_*i*_|, |*C*_*j*_| represent the cardinality of the set *C*_*i*_ and the cardinality of the set *C*_*j*_, respectively, with |*C*_*i*_|>|*C*_*j*_|.

### Chunks replacement (Fig 1d)

**Fig 2:**
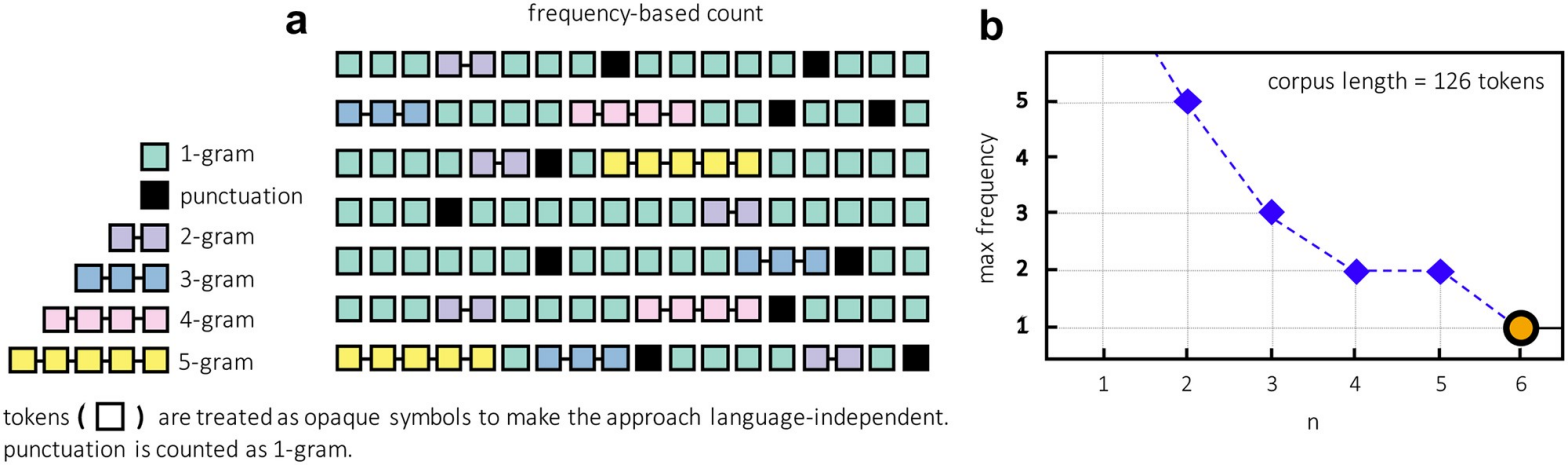
Chunking. Visual representation of the chunking process: (**a**) identification of repeated n-grams and (**b**) max frequency values over *n*.

This step uses the cleaned list (from the previous step) to replace the final chunks into the initial corpus. Fig 2 shows the overall chunking process with a visual representation. In other words, the chunk replacement step takes each chunk from the list of chunks from the previous cleaning step and replaces it within the raw corpus using a replacement of spaces (“”) with any other non-alphanumeric character not present in the raw corpus (for instance, the underscore symbol “_“). For example, the 3-gram “the current account” is replaced by “the_current_account.”

### Pseudo-labeling (Fig 1e)

This step uses chunks for labeling and comparing them with those in the annotated corpus.

The proposed methodology does not directly provide labels to be compared with those in the annotated corpus for evaluating the chunking results. For this reason, pseudo-labels are generated. Pseudo-labels are created for each token by assigning one of the BIO labels. For example, the tokens of the chunk “the current account deficit” will have the following pseudo labels: [the]B [current]I [account]I [deficit]I, or the chunk “will narrow” will have the following labels per token [will]B [narrow]I. This is useful for evaluating the proposed method with annotated corpora that use BIO labels, such as the CoNLL-2000 Corpus [12] and the Alan Ritter Twitter Corpus [67]. Examples of these pseudo-labels are shown in Table 3, whereas examples of BIO labels are shown in Table 4.

**Table 3:** Unsupervised paradigm for the attribution of pseudo-labels.

Unsupervised paradigm	
*input*	He reckons the current account deficit
*output*	[He] [reckons] [the current account deficit]
*pseudo* − *labels*	[He]B [reckons]B [the]B [current]B [account]I [deficit]I

The PoS of each token is ignored in the unsupervised chunker.

**Table 4:** Traditional paradigm for the attribution of BIO labels.

Supervised and Semi-supervised paradigm	
*input*	He reckons the current account deficit
*output*	[He]B_NP_ [reckons]B_VP_ [the]B_NP_ [current]I_NP_ [account]I_NP_ [deficit]I_NP_
*BIO* *labels*	[He]B_NP_ [reckons]B_VP_ [the]B_NP_ [current]I_NP_ [account]I_NP_ [deficit]I_NP_

The output comes with BIO-labels in both supervised and semi-supervised chunkers.

As previously mentioned, this method aims to obtain text chunks without providing an indication of the PoS. Hence, a pseudo-label for “B,” “I,” and “O” can be assigned, although the internal role of each token (PoS annotation) is not provided. Both corpora used for the evaluation (Penn Treebank CoNLL-2000 for newswire articles and Alan Ritter Corpus for Twitter text data) provide a BIO labeling schema.

### Evaluation (Fig 1f)

This last step provides evaluation metrics, such as the *F*_*β* = 1_ score, to assess the accuracy of the method in different types of corpora. Following the traditional choice of evaluation for a text chunking task, the accuracy is calculated by comparing the BIO labels of the predicted chunks (pseudo-labels in this case) with the BIO labels of the original chunks using the *F*_*β* = 1_ score [68, 69]:

$$\begin{array}{c} F_{\beta }=\left(1+\beta^{2}\right)\cdot \frac{P\cdot R}{{(\beta }^{2}\cdot P)+R} \end{array}$$ (8)

$$\begin{array}{c} {\;F}_{\beta =1}\;=2\cdot \frac{P\cdot R}{P+R} \end{array}$$ (9)

$$\begin{array}{c} Precision\;\left(P\right)=\frac{true\;positives}{true\;positives+false\;positives} \end{array}$$ (10)

$$\begin{array}{c} Recall\;\left(R\right)=\frac{true\;positives}{true\;positives+false\;negatives} \end{array}$$ (11)

where *precision* *P* is the percentage of chunks that are correct and *recall* *R* is the percentage of chunks that were found by the unsupervised text chunker; in addition, *true positives* is the number of tokens that have been correctly predicted as “B” (beginning of a chunk), *false positives* is the number of tokens that are incorrectly predicted as the begin of a chunk “B,” and *false negatives* indicates the number of tokens that are not predicted as “B” although they actually are at the begin of a chunk. It is worth noting that the proposed unsupervised approach does not naturally provide labels (predictions) to calculate *F*_*β*_, *P*, and *R*. Nevertheless, to conduct a comparison with existing approaches for text chunking, which are mostly based on labeled datasets (similar to the annotated corpora used in this study), pseudo-labels are generated, as described previously in this section. Hence, the predictions are the pseudo-labels and the true labels are the annotated labels.

## Results and discussion

Previous studies evaluating supervised learning methods have typically shown a high accuracy. These results have generally been obtained by training and testing models using an annotated treebank. However, this high accuracy degrades when the same model is applied to text from a different domain. This has increased and directed the attention toward unsupervised approaches. In this study, textual chunks were obtained using a completely unsupervised approach based on the frequency of n-grams combined with automated pre-processing and cleaning steps.

**Fig 3:**
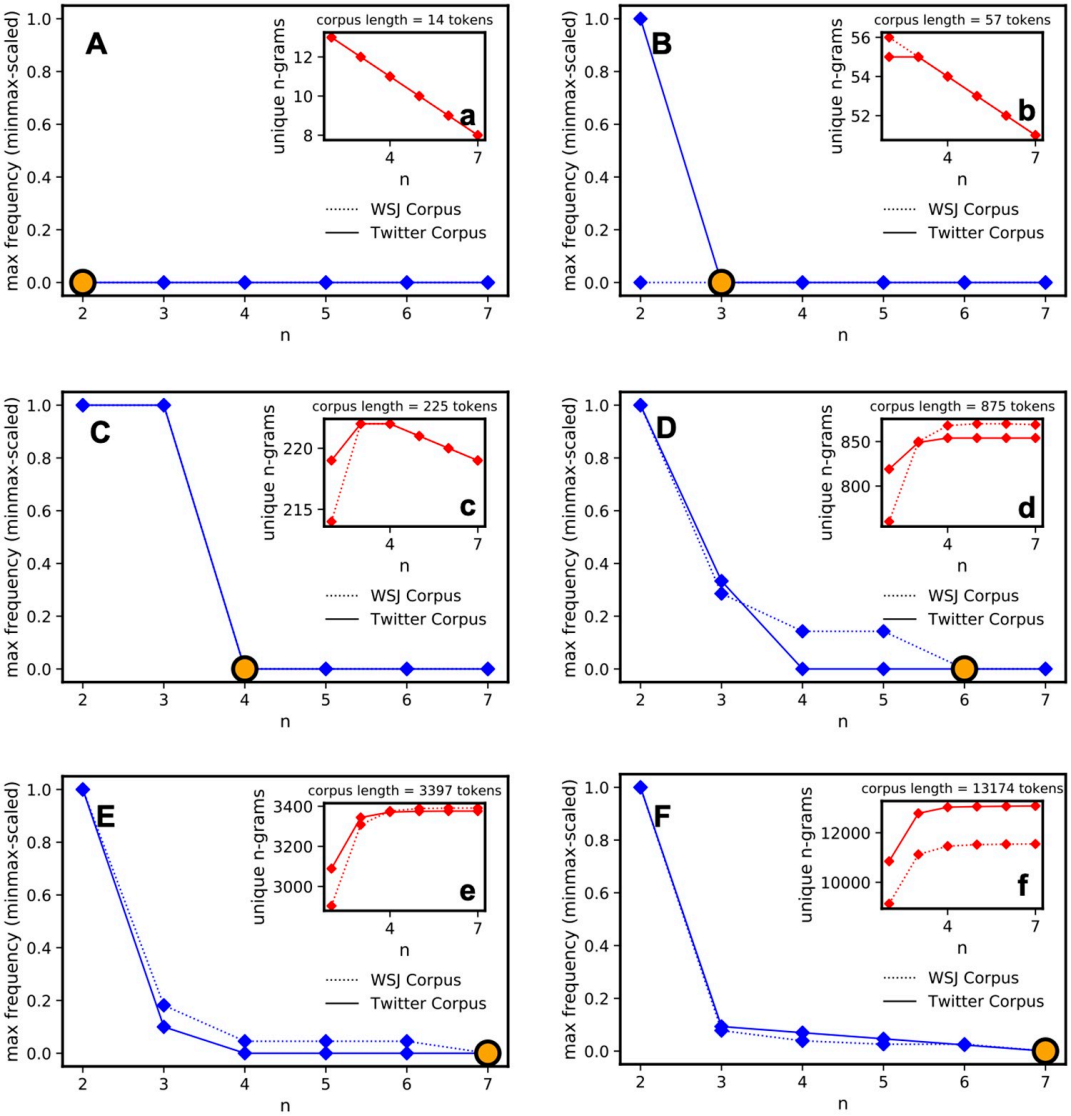
Maximum frequency of chunks varying the corpus length. Exploration of dependencies between the maximum frequency of n-grams and the length of the input corpus in terms of the number of tokens.

The frequencies of different n-grams are calculated by varying the corpus length expressed as the number of tokens. Fig 3 shows an example in which the length of the corpus has been varied (with six different lengths). The length is indicated at the top-left of each plot. The larger graph (with blue data points) shows the value of *n* along the x-axis, and the n value and maximum frequency of the n-grams at each *n* are shown along the y-axis. When the length of the corpus increases, the value of *n* at the maximum frequency (where *F* > 1) also increases. Beyond this value of *n*, there is a frequency convergence at 1, meaning that there are no repeated n-grams after that value.

In the smaller graph (upper-right corner), the numbers of unique n-grams are plotted as a function of *n*. It is possible to see how this number decays when *n* increases in a small corpus. This is because, with a large *n* and a relatively small corpus, many n-grams cannot be generated, i.e., there is a limited number of available tokens. By increasing the size of the corpus, it can be seen that the value stabilizes and sets on a certain number because n-grams are not repeatedly found above a specific value of *n*, as shown by the yellow dot.

This initial exploration helps calculate the maximum value of *n* and provides insight into the type of input corpus required to build the n-grams. An unsupervised approach has been applied to two different corpora: a newswire corpus and a social media text corpus. The choice of these two different datasets allows the proposed approach to be compared to two different types of textual styles: news and social media. The first is a more conventional type of text, whereas the second is an informal type of text in which grammatical rules may be broken. The newswire corpus is part of the English Penn Treebank II (sections 15-16-17-18-20) consisting of Wall Street Journal news [12, 70]. It has been extensively used for supervised text chunking in the literature, as previously mentioned in the introduction. The social media corpus is the Twitter corpus proposed by Alan Ritter [67].

**Fig 4:**
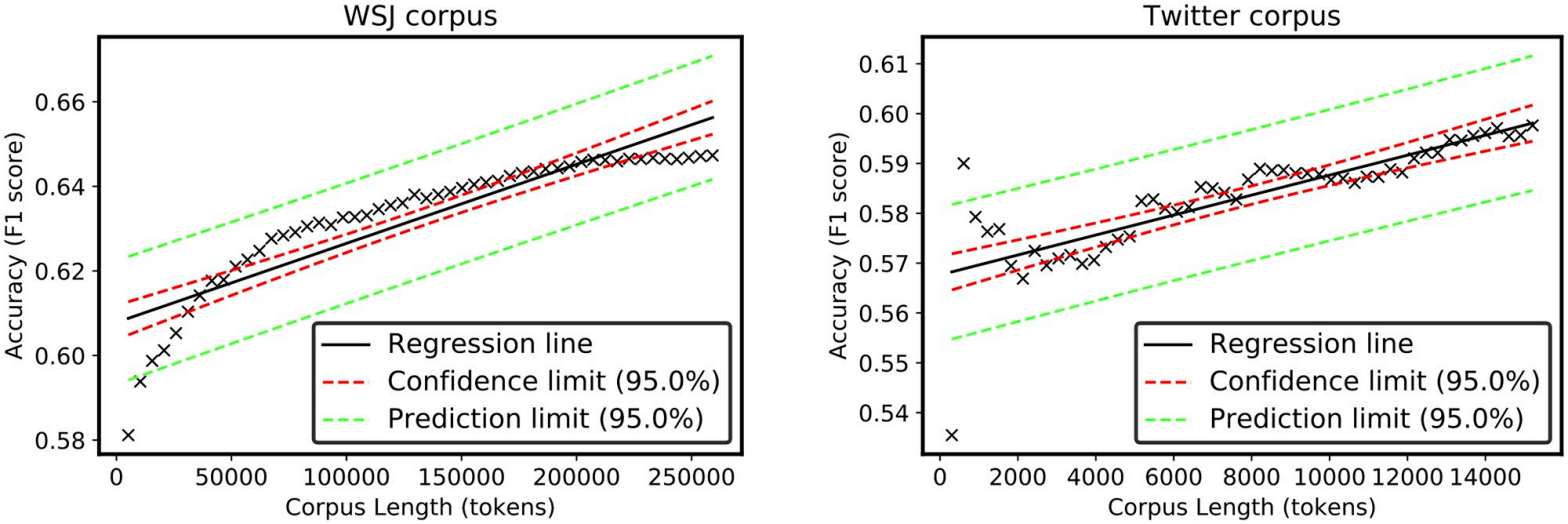
Linear fit of the two sets of data points. Regression line for both analyzed corpora, with the WSJ corpus on the left and Twitter corpus on the right.

**Fig 5:**
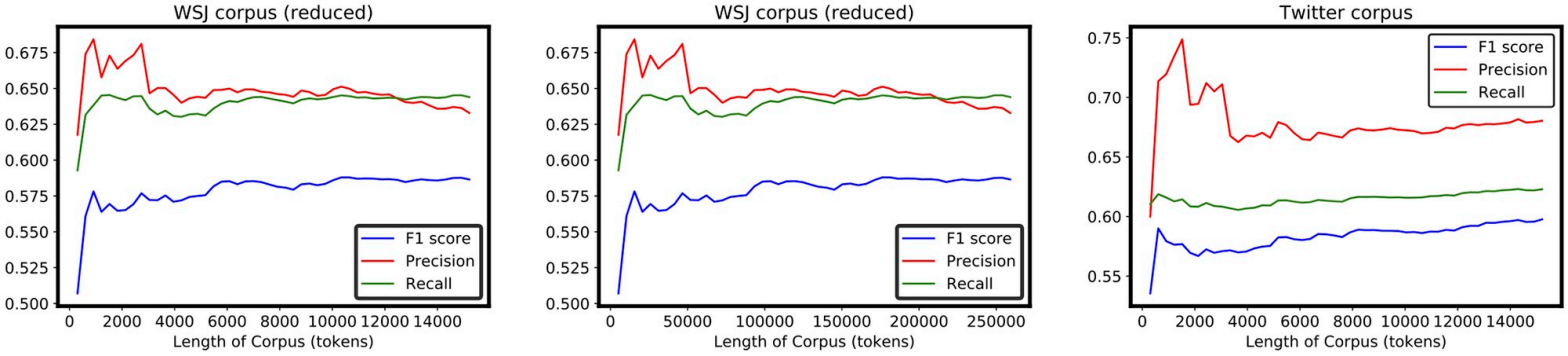
Accuracy, precision, recall. The *F*_*β* = 1_ score, precision, and recall as a function of the corpus length. In this graph, the WSJ corpus length has also been reduced to the same dimension as the Twitter Corpus.

Fig 4 shows two graphs in which the accuracy of the proposed method (measured using *F*_*β* = 1_) on the two different corpora is plotted over the number of non-unique tokens. The left side contains the graph for the WSJ corpus, whereas the right side contains the results for the Twitter corpus. As can be seen, with an increasing number of tokens, the accuracy in both corpora increases, as might be expected, because if more data are available more frequent chunks are considered to be counted. However, a striking difference was noted between the two increasing rates. The accuracy (*F*_*β* = 1_ score) with the Twitter corpus increases with a linear tendency. By contrast, the WSJ corpus shows a reluctance in increasing linearly after a certain number of tokens. In fact, the left graph of Fig 5 shows a point after which the precision (for the WSJ corpus) reaches a plateau with an increase in the number of tokens. This behavior was also registered with a reduced WSJ corpus using a comparable number of tokens as the Twitter Corpus length (central graph in Fig 5).

To properly assess the linearity of the graphs, a linear model was fitted to the data points. Using this approach, the accuracy on the Twitter corpus showed a more linear increase compared to that of the WSJ corpus, which showed a logarithmic tendency. These results suggest that the proposed unsupervised method for text chunking may provide better results when applied to Twitter data compared with the newswire corpora. The accuracy of the chunker on the newswire corpus had a logarithmic tendency rather than a linear tendency, providing a risk of an asymptotic convergence to a higher accuracy. This may suggest an inefficient behavior if the newswire corpus is used recursively. Both graphs of Fig 4 show an increase in accuracy as the size of the corpus increases. For the WSJ corpus, an initial rapid and regular increase in accuracy can be seen. For the Twitter corpus, the method achieves a more fluctuating accuracy at the beginning and then grows uniformly afterward. Fig 4 shows a linear generalization of the accuracy trend. It can be seen that on the Twitter corpus the unsupervised method achieves a linear growth in performance with the number of input tokens, whereas for the WSJ corpus, a linear fitting seems to be inadequate. This corpus presents a logarithmic trend with a tendency to converge toward a higher accuracy. This result may favor unsupervised text chunking models for social media texts in this specific case of Twitter, in which the language often does not strictly follow pre-defined grammar/syntax rules. The proposed method based on n-gram frequencies is completely independent from the syntax and grammar rules that govern the input language. Table 5 summarizes the best results obtained with the proposed method on these two different corpora. In addition, Table 6 shows the differences between state-of-the-art approaches that apply these two standard annotated data-sets for text chunking. The focus is mainly on two aspects: (1) the substantial use of the WSJ newswire corpus (CoNLL-2000), and (2) the direction of the approaches presented in the literature toward methodologies that include at least semi-supervision (S-SUP) during the learning process. Consequently, the *F*_*β* = 1_ score obtained by the semi-supervised methods (S-SUP) is quite high (see Table 2). However, among an extremely small number of unsupervised methods (UNS), the corpus developed by Ponvert et al. achieved an *F*_*β* = 1_ score and precision *P* slightly higher than those obtained in the present study. By contrast, in their paper [49], the recall turns out to be slightly lower (*R* = .639) than that found with our approach (*R* = .684). Despite this, the unsupervised method proposed herein (proposed UNS) is the only method that has been applied to Twitter (among those testing the method on these two standard corpora). This represents a novelty, and highlights a gap, in the literature. It is important to note that the results of these annotated corpora are not yet competitive with the accuracy of existing supervised learning studies. Unsupervised learning of natural language is still a challenge for NLP and other fields where the aim is to learn the rules underlining sequential data in an unsupervised fashion. In addition, the application of such approaches to sequential information differing from written natural language can be a challenge. For instance, as stated by Febres et al. [54], whereas a space in natural language (i.e., the English language) is used as delimiter, this may not be true with other symbolic data. In such situations, the adoption of algorithms such as the fundamental scale algorithm [56] based on a minimization of the overall entropy can be a worthwhile direction. Moreover, incorporating symbolic diversity and symbolic entropy in unsupervised approaches, as with the study by Febres et al. [53], can be worth exploring when comparing different symbolic sequential data.

**Table 5:** Comparison of accuracy for the highest number of tokens available.

Corpus	*F*_*β*=1_	P	R
WSJ—Penn English Treebank	.64729	.67278	.68463
Twitter Corpus (Alan Ritter)	.59762	.68037	.62291

**Table 6:** Comparison of *F*_*β* = 1_ with state-of-the-art methods.

	WSJ Corpus		Twitter Corpus	
	S-SUP	UNS	S-SUP	UNS
Peters et al. [22]	.963	-	-	-
Clark et al. [32]	**.967**	-	-	-
Ritter et al. [67]	.854	-	**.867**	-
Ponvert et al. [49, 58]	-	**.698**	-	-
**Proposed UNS**	-	.647	-	**.597**

## Conclusion

In this study, an unsupervised approach to text chunking was introduced as one of the basic tasks in NLP. A methodology for extracting chunks in an unsupervised fashion was presented as an alternative solution to applications in which a labeled treebank is unavailable.

In this study, the presented methodology for unsupervised text chunking was compared using two different corpora: a newswire corpus composed of Wall Street Journal articles and a Twitter corpus of random tweets. Both corpora have manual chunk annotations identified by human experts. These corpora are typically used for evaluating supervised and semi-supervised text chunkers. This provides a means to evaluating the proposed unsupervised method using accuracy metrics. In fact, a proper pseudo-labeling step was conducted, converting an unlabeled output into a labeled one. To measure the accuracy with the *F*_*β* = 1_ score, chunks are converted in such a way that pseudo-labels assigned to each token can be compared with the original labels of tokens in both corpora.

The accuracy of the unsupervised chunking method was compared between the news-wire corpus and the Twitter corpus. For the Twitter corpus, the *F*_*β* = 1_ score followed a linear trend with an increase the number of tokens (the length of the corpus). The precision (*P*) and recall (*R*) also showed a similar dynamic. The news-wire corpus presented the opposite behavior, the accuracy of which did not follow the same tendency. The annotations upon which the accuracy was measured are based on a syntax schema; hence, it can be interpreted that the WSJ corpus conveys more complex syntactical rules than the Twitter corpus. The limited number of characters per tweet (140 characters) and the informal way in which people communicate through online posts in comparison to the language used in WSJ news articles may play a role in favoring an unsupervised chunker for Twitter.

The proposed chunker is based on a simple mechanism of counting repeated occurrences of n-grams, which is an increasingly more effective mechanism when more data become available. The log-linear tendency found by Pitler et al. [71] on their web news corpus is also evident in the WSJ corpus. However, it is worth noting that the problem of the asymptotic tendency of a log-linear function of the accuracy (evident in the WSJ corpus) can lead to the consequential risk of convergence.

The performance of this method was measured based on the *F*_1_ score. The accuracy of this method for two different types of text was shown to increase gradually when increasing the number of tokens as an input. This approach has demonstrated the potential to be more accurate with recursive usage. The more un-labeled data that are provided, the greater the chances of capturing new repeated n-grams, which are chunk candidates. This is in line with the recurrent idea that “more data are better data,” indicating that the more chunks the algorithm can obtain for its training the better it will perform. Nonetheless, the quality of these chunks and the type of text where the chunks come from are as important for acquiring more accurate results in unsupervised chunking methodologies. For instance, in the study by Pitler et al. [71], a web-scale n-gram model proved that the accuracy of noun phrase identification increase the log-linearly when more data are available. The datasets used for the evaluations are an important aspect to consider. For instance, the results reported in this paper refer to datasets designed to apply supervised methodologies with the support of different labels. In the presented case, during the application of the algorithm, the labels were ignored. However, these labels were then used in the evaluation phase. Hence, the application of this method can be extended to any type of sequential data. By contrast, the evaluation phase presented here requires the presence of annotations in the corpus, which can be non-trivial in finding non-textual sequential data. Furthermore, a type of labeling (and pseudo-labeling) schema differing from the conventional labels of the treebanks may be required.

Future studies will test the method on other types of sequential data. It is worth noting that human annotations may contain biases. Consequentially, measuring the accuracy on these labels can lead to biased conclusions. This bias is intrinsic in the measurement error and subjectivity of human annotation. However, attempts have been made to minimize this bias by using gold treebanks, in which annotations have been made by domain experts. The presence of new languages that are created by online users may not reflect the standards used by domain experts. In the unsupervised approach presented herein, the learning task for a machine does not occur with the supervision of a human expert, but with a repetition-based approach. This approach, for certain aspects, reflects a similarity with the learning process of new languages by children. Like Skinner’s empiricist theory of language acquisition, other languages, encoded with sequential data, can be learned by a machine with a repetition-based strategy. The lack of experimentally measured precision in the results may be metaphorically linked to the absence, for a machine, of the innate faculty of the human brain in the acquisition of language, as theorized by Chomsky.
