# Correction: Unsupervised acquisition of idiomatic units of symbolic natural language: An n-gram frequency-based approach for the chunking of news articles and tweets

**DOI:** 10.1371/journal.pone.0245404

**Published:** 2021-01-07

**Authors:** Dario Borrelli, Gabriela Gongora Svartzman, Carlo Lipizzi

The image for Fig 5 incorrectly shows the left line-plot and the middle line-plot as same. Please see the correct Fig 5 here.

**Fig 5:**
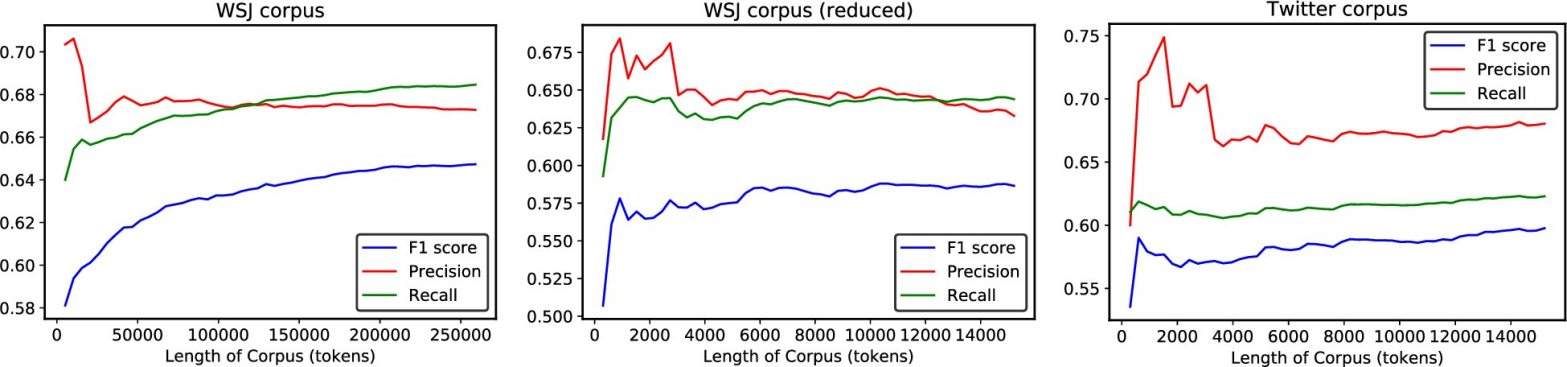
Accuracy, precision, recall. The *F*_***β* = 1**_ score, precision, and recall as a function of the corpus length. In this graph, the WSJ corpus length has also been reduced to the same dimension as the Twitter Corpus.
